# Effects of Hydrogel-to-Oleogel Ratio and Potato Protein Isolate Concentration on the 3D Printing and Post-Heating Properties of Food-Grade Bigels

**DOI:** 10.4014/jmb.2607.07038

**Published:** 2026-09-04

**Authors:** Seri Park, Jiho Sohn, Yourim Oh, Jin-Kyu Rhee

**Affiliations:** 1 Department of Food Science and Biotechnology, Ewha Womans University, Seoul 03760, Republic of Korea; 2 SuFAB Inc., 123 Digital-ro 26-gil, Guro District, Seoul 04790, Republic of Korea

**Keywords:** Bigel, Potato protein isolate, 3D food printing, Dimensional fidelity, Post-heating stability

## Abstract

This study investigated the effects of the hydrogel-to-oleogel ratio and potato protein isolate (PPI) concentration on the printing and post-heating properties of food-grade bigels. Bigels with hydrogel-to-oleogel ratios of 10:0, 7:3, 5:5, 3:7, and 0:10 were evaluated for physical stability, rheological behavior, structural recovery, and 3D printing performance. Among the biphasic formulations, H_7_O_3_ exhibited the highest liquid-binding capacity (99.43%) and structural recovery ratio (88.7%) and produced lateral dimensions closest to those of the CAD model. H_7_O_3_ was therefore selected for PPI incorporation at 0-10 wt%. PPI incorporation improved the initial dimensional fidelity of the printed constructs and reduced rheological softening during heating. After heating at 120°C for 10 min, the 0% PPI construct collapsed, whereas all PPI-containing constructs retained identifiable three-dimensional shapes. Post-heating lateral deformation decreased with increasing PPI concentration, and the 10% PPI formulation exhibited width and length retention values closest to 100%. In addition, the 7 and 10% PPI formulations showed significantly lower cooking loss than the 3 and 5% formulations. These findings demonstrate that the hydrogel-to-oleogel ratio established the printing characteristics of the bigels, whereas PPI concentration modulated their initial dimensional fidelity and post-heating stability. The developed bigels have potential as 3D-printable structured lipid systems for fat-replacement applications.

## Introduction

Fat plays important structural and sensory roles in meat and meat analogues by contributing to texture, juiciness, mouthfeel, and flavor release. However, replacing animal fat with liquid vegetable oils remains challenging when defined fat-like domains are required because unstructured oils generally lack the solid-like integrity needed to maintain their shape and position within a food matrix. They may flow or migrate during processing and therefore cannot readily provide the structural characteristics associated with solid animal fat. For such applications, vegetable oils can be structured into semisolid materials that are more readily shaped and retained within a food product [1]. Extrusion-based three-dimensional (3D) food printing may further process these materials into reproducible structured-fat components through layer-by-layer deposition according to a digital design, providing a potential basis for their application in thermally processed meat analogues [2].

Bigels are biphasic semisolid systems composed of hydrogel and oleogel phases and provide a means of combining aqueous and lipid components within a single material [3]. In the present base formulation, the hydrogel phase consisted of waxy corn starch, κ-carrageenan, and gellan gum, whereas the oleogel phase was prepared from sunflower oil and candelilla wax. The polysaccharide combination was used to provide viscosity and the structural support required for extrusion and layer deposition, while candelilla wax structured sunflower oil into a semisolid oleogel phase [4, 5]. Together, these phases were intended to form a printable matrix containing structured plant oil. In this configuration, the hydrogel phase provides an aqueous structural network, whereas the oleogel phase immobilizes liquid vegetable oil within a semisolid lipid matrix, allowing the bigel to function as a plant-based fat-like material with both printability and structural integrity [5, 6].

The rheological and 3D printing properties of bigels are strongly influenced by the hydrogel-to-oleogel ratio. For example, Qiu *et al*. [7] reported that varying the oleogel fraction in bigels composed of a κ-carrageenan–xanthan gum hydrogel and a beeswax–corn oil oleogel affected their rheological behavior, structural recovery, and printability. Fernandes *et al*. [8] similarly reported oleogel-fraction-dependent changes in the rheological and printing properties of bigels prepared with agar–xanthan gum or gelatin–xanthan gum hydrogels and a sunflower oil–beeswax oleogel. Adjustment of this ratio can alter physical stability, viscoelasticity, structural recovery, extrusion behavior, and dimensional fidelity, thereby determining the balance between flow through the nozzle and resistance to deformation after deposition [7, 8]. However, a printed structured-lipid construct intended for use in a cooked product represents an intermediate rather than a final structure. During heating, the hydrogel and oleogel phases may undergo different temperature-dependent changes, potentially disturbing the structural balance established at room temperature. Consequently, a formulation that exhibits smooth extrusion and high initial shape fidelity may still undergo dimensional deformation, structural collapse, or material loss during cooking [9]. Initial printability alone therefore does not establish whether a 3D-printed bigel can retain its intended structure during subsequent thermal processing.

A potential approach to improving post-heating stability is to introduce heat-responsive functionality into the hydrogel phase. Potato protein isolate (PPI) was selected because potato proteins undergo thermal unfolding and aggregation within food-relevant temperature ranges and can develop elastic heat-set structures [10]. Potato protein–gellan gum systems have also exhibited composition-dependent changes in mechanical properties [11], suggesting that PPI may modify the thermal response of the present gellan gum-containing hydrogel. PPI was therefore incorporated into the hydrogel phase as a heat-responsive component. Although PPI may also influence the initial rheological and 3D printing properties of the resulting bigel, its heat-induced aggregation was expected to provide additional structural resistance during post-print heating. However, it remains unclear whether this rheological response can be translated into improved dimensional stability and reduced overall cooking loss in actual 3D-printed bigel constructs.

Accordingly, this study investigated the effects of hydrogel-to-oleogel ratio and PPI concentration on the initial 3D printing and post-heating properties of food-grade bigels. Bigels with different phase ratios were first characterized to identify a base formulation with an appropriate balance of physical stability, rheological behavior, structural recovery, and dimensional fidelity. Different concentrations of PPI were then incorporated into the hydrogel phase of the selected formulation to determine their effects on heating-cooling rheology and printing feasibility. The 3D-printed constructs were subsequently thermally processed to assess dimensional changes and overall cooking loss. It was hypothesized that adjustment of the hydrogel-to-oleogel ratio would establish a printable base formulation, whereas PPI concentration would modulate both the initial 3D printing behavior and heat-induced structural reinforcement, thereby affecting the post-heating stability of the 3D-printed bigels.

## Materials and Methods

### Materials

κ-Carrageenan was purchased from Sigma-Aldrich (USA). Waxy corn starch and potassium chloride were obtained from Samyang Corporation and Daejung Chemical & Metals Co., Ltd., respectively (Republic of Korea). Gellan gum and calcium chloride were purchased from ES Food Co., Ltd. and Samchun Pure Chemical Co., Ltd., respectively (Republic of Korea). Candelilla wax and sunflower oil were obtained from Fresh Farm and Herbnoori, respectively (Republic of Korea). Potato protein isolate (PPI; VegeWhip, SaporePuro, Italy; country of origin: the Netherlands) contained 93.2% protein, 0.1% fat, <0.1% carbohydrate, and 0.2% dietary fiber. According to the supplier’s specifications, its moisture and ash contents were ≤9 and ≤5%, respectively.

### Preparation for Bigels

To prepare the hydrogel phase, waxy corn starch (WCS, 4 wt%, based on the total weight of the hydrogel phase) was dispersed in distilled water and heated at 105°C under continuous stirring for 1 h. κ-Carrageenan (KC, 0.75 wt%) and potassium chloride (KCl, 0.75 wt%) were subsequently added, and the dispersion was stirred at 95°C for an additional 1 h. Gellan gum (GG, 2 wt%) and calcium chloride (CaCl_2_, 0.2 wt%) were then added, and the mixture was continuously stirred at 95°C until a homogeneous dispersion was obtained. The resulting hydrogel was cooled and stored at 4°C overnight.

To prepare the oleogel phase, candelilla wax was added to sunflower oil at a concentration of 10 wt%, based on the total weight of the oleogel phase. The mixture was heated at 80°C under continuous stirring until the candelilla wax was completely melted and uniformly dispersed. The resulting oleogel was then cooled to room temperature.

Before bigel preparation, the hydrogel and oleogel phases were equilibrated to room temperature. The two phases were combined at hydrogel-to-oleogel weight ratios of 10:0, 7:3, 5:5, 3:7, and 0:10 and homogenized at 11,000 rpm for 5 min using a high-speed homogenizer (Polytron PT2100, Kinematica, Switzerland). The resulting bigels were designated H_10_O_0_, H_7_O_3_, H_5_O_5_, H_3_O_7_, and H_0_O_10_, where H and O represent the relative proportions of the hydrogel and oleogel phases, respectively. All bigel samples were stored at 4°C until further analysis.

### Preparation of PPI-Containing Bigels

The polysaccharide hydrogel was prepared according to the procedure described in Bigels subsection and cooled to room temperature before PPI incorporation. PPI was added at concentrations of 0, 3, 5, 7, and 10 wt% based on the weight of the prepared polysaccharide hydrogel. The mixtures were homogenized at 11,000 rpm for 2 min using a high-speed homogenizer (Polytron PT2100, Kinematica). The resulting PPI-containing hydrogels were stored at 4°C overnight to allow hydration. After equilibration to room temperature, each PPI-containing hydrogel was combined with the oleogel phase and homogenized at 11,000 rpm for 5 min, as described in Bigels subsection. The resulting samples were stored at 4°C until further analysis.

### Liquid Binding Capacity

A 10 g portion of each sample was placed in a 15 mL centrifuge tube and stored at 4°C for 24 h. Before centrifugation, the samples were equilibrated at room temperature for 1 h. The samples were then centrifuged at 11,000 rpm and 25°C for 15 min using a 1696R centrifuge (Labogene, Republic of Korea). Following centrifugation, each tube was inverted onto filter paper to remove the separated liquid. The weight of the remaining sample was then measured. The liquid-binding capacity (LBC) was calculated using Equation: The liquid binding capacity (LBC) was calculated using Equation:

$$LBC\left(\%\right)=\frac{W_{f}}{W_{i}}\times 100$$

where *W_i_* and *W_f_* represent the sample weights before centrifugation and after removal of the separated liquid, respectively. All measurements were performed in triplicate.

### FT-IR Spectroscopy

FT-IR spectra of the samples were recorded using a Cary 630 FT-IR spectrometer equipped with a diamond attenuated total reflectance (ATR) accessory (Agilent Technologies, Santa Clara, CA, USA). The spectra were collected over the wavenumber range of 4000–650 cm^-1^ at a resolution of 4 cm^-1^ and processed using MicroLab PC software.

### Rheological Measurements

Rheological measurements were performed using an MCR 102 rheometer (Anton Paar, Austria) equipped with a parallel-plate geometry with a diameter of 25 mm and a gap of 2 mm. A thin layer of silicone oil was applied around the exposed edge of each sample to minimize moisture evaporation. Before measurement, the samples were allowed to rest for 10 min to permit structural relaxation. Rheological data were analyzed using RheoCompass software (version 1.22).

Strain sweep tests were conducted at 25°C and a constant frequency of 1 Hz over a strain range of 0.001–10% to determine the linear viscoelastic region (LVR). The yield stress was determined as the crossover point where the storage modulus (G′) equaled the loss modulus (G″).

Frequency sweep tests were conducted over a frequency range of 0.01–10 Hz at a strain of 0.1%, which was within the LVR. G′ and G″ were recorded, and the complex modulus (G*) was calculated from these values. The frequency dependence of G′ was evaluated from the slope of the linear regression of log G′ against log frequency.

Steady-shear flow measurements were performed at 25°C by increasing the shear rate from 0.01 to 100 s ¹. The resulting apparent viscosity data were fitted to the following power-law model:

$$\eta =K\gamma^{\cdot n-1}$$

where *η* is the apparent viscosity (Pa·s), $\dot{\gamma }$ is the shear rate (s^-1^), *K* is the consistency coefficient (Pa·s^n^), and *n* is the flow behavior index.

A three-interval thixotropic test (3ITT) was performed at a frequency of 1 Hz to evaluate structural recovery after large deformation. The samples were subjected to a low strain of 0.01% for 2 min, followed by a high strain of 100% for 2 min and a low strain of 0.01% for 4 min. The recovery ratio was calculated using Equation:

$$\text{Recovery ratio (\%) =}\frac{{G^{\prime }}_{3rd,end}}{{G^{\prime }}_{1st,end}}\times 100$$

where *G*′*_1st, end_* and *G*′*_3rd, end_* represent the storage modulus values measured at the ends of the first and third intervals, respectively.

Temperature sweep tests were performed at a strain of 0.01% and a frequency of 1 Hz, both within the LVR, to evaluate the temperature-dependent viscoelastic properties of the bigels. The samples were balanced at 25°C for 10 min and subsequently heated from 25 to 80°C at a rate of 2°C/min. G′ and G″ were recorded as functions of temperature. The complex shear modulus (G*) and loss tangent (tan δ) were calculated from these values using the following equations:

$$\begin{array}{l} G*=\sqrt{{\left(G^{\prime }\right)}^{2}+{\left(G^{\prime \prime }\right)}^{2}}\\ \tan\delta =\frac{G^{\prime \prime }}{G^{\prime }} \end{array}$$

### 3D Printing Performance

The 3D printing performance of the bigel samples was evaluated using a screw-based 3D food printer (CGBIO, South Korea). A rectangular prism model measuring 15.0 × 15.0 × 10.0 mm was designed using 3D CAD software (Inventor, USA) and exported in STL format. The STL file was converted into G-code using slicing software (Simplify3D, version 4.1.1, USA). The samples were printed at room temperature through an 18G stainless-steel nozzle. The 3D printing speed and extrusion rate were both set at 400 mm/min. Immediately after 3D printing, the width, length, and height of each printed construct were measured using a digital caliper. The dimensional fidelity of the bigels with different hydrogel-to-oleogel ratios was assessed by comparing the measured dimensions with the corresponding dimensions of the CAD model.

For the PPI-containing bigels, the width, length, and height of each printed construct were measured immediately after printing. The dimensional accuracy of each printed construct relative to the corresponding dimension of the CAD model was calculated using the following equation:

$$\text{Dimensional accuracy (\%) =}1-\left|\frac{D_{measured}-D_{designed}}{D_{designed}}\right|\times 100$$

The oven (COV23K, Guangdong Chengying Electrical Appliances Co., Ltd., China) was preheated to 120°C for 10 min. The printed constructs were then heated at 120°C for 10 min and subsequently cooled to room temperature before further measurements. Post-heating dimensional stability was evaluated by comparing the width, length, and height of the same printed construct before and after heating. The retention of each dimension was calculated using Equation:

$$\text{Dimensional retention (\%) =}\frac{\text{Dimension after heating}}{\text{Dimension before heating}}\times 100$$

### Cooking Loss

The initial weight of each 3D-printed PPI-containing construct was recorded before thermal processing. The oven was preheated to 120°C for 10 min. The constructs were heated in an oven at 120°C for 10 min and subsequently cooled to room temperature. After cooling, the exuded liquid was removed, and the remaining portion of each construct was weighed to determine the final weight. Cooking loss was calculated using equation:

$$\text{Cooking loss (\%)}=\frac{\text{Weight before heating-Weight after heating}}{\text{Weight before heating}}\times 100$$

### Statistical Analysis

All measurements were performed in triplicate, and the results were expressed as the mean ±standard deviation. Statistical analysis was performed using one-way analysis of variance, followed by Duncan’s multiple range test, using SPSS software (version 12.0, SPSS Inc., USA). Differences were considered statistically significant at *p* < 0.05. Graphs were generated using SigmaPlot software (version 12.5, Systat Software, USA).

## Results and Discussion

### Effects of Hydrogel-to-Oleogel Ratio on the Properties of Bigels

**Physical stability.** The vial inversion test and LBC measurements were used to evaluate the physical stability of the samples. All samples remained self-standing, with no visible macroscopic phase separation or liquid leakage (Fig. 1A). In addition, all formulations exhibited LBC values above 90% (Fig. 1B), indicating a high overall capacity to retain the incorporated liquid components under centrifugal force. Among the biphasic bigel formulations, H_7_O_3_ exhibited the highest LBC value of 99.43%, and LBC subsequently decreased as the oleogel proportion increased. This trend indicates that increasing the oleogel fraction reduced the ability of the biphasic matrix to retain its liquid components under centrifugal force.

**FT-IR properties.** The FT-IR spectra of samples with different hydrogel-to-oleogel ratios are presented in Fig. 2. In the hydrogel-containing samples, the broad absorption band in the range of 3700–3000 cm^-1^ was attributed to O–H stretching vibrations associated with water and hydroxyl groups in the hydrogel matrix [12, 13]. The band near 1650 cm^-1^ was assigned to the H–O–H bending vibration of water [14]. The oleogel-containing samples exhibited characteristic bands at approximately 2920 and 2850 cm^-1^, corresponding to the asymmetric and symmetric stretching vibrations of aliphatic C–H groups, respectively [15]. Additional bands near 1740, 1460, and 1160 cm^-1^ were assigned to ester C=O stretching, CH_2_ bending, and ester C–O stretching vibrations, respectively [15]. The spectra of the bigels displayed the characteristic absorption bands of both the hydrogel and oleogel phases. No new absorption bands or appreciable shifts in the major characteristic peaks were observed. These spectral features were consistent with the physical combination of the two phases, with no FT-IR detectable evidence of new covalent bond formation during bigel preparation [12, 16].

**Rheological behavior and structural recovery.** The rheological properties of the samples with different hydrogel-to-oleogel ratios were evaluated using strain sweep, frequency sweep, steady-shear flow, and 3ITT (Fig. 3). Within the LVR, G′ and G″ remained relatively constant, with G′ exceeding G″ in all samples (Fig. 3A). Beyond the LVR, both moduli decreased, indicating progressive disruption of the gel structure under increasing deformation [5]. The hydrogel-rich samples generally exhibited higher moduli and appeared to exhibit broader LVRs than the oleogel-rich samples, suggesting greater resistance to deformation.

Throughout the tested frequency range, G′ remained higher than G″ without a crossover, indicating solid-like viscoelastic behavior (Fig. 3B) [6]. Both moduli exhibited relatively weak frequency dependence, and the fitted slopes of log G′ versus log frequency did not differ significantly among the samples (*p* > 0.05; Table 1). These results indicate that the frequency dependence of G′ was similar among the formulations within the tested range.

The apparent viscosity of all samples decreased with increasing shear rate, demonstrating shear-thinning behavior (Fig. 3C). Apparent viscosity generally decreased as the oleogel proportion increased. This shear-thinning behavior may facilitate extrusion by reducing flow resistance at the high shear rates encountered during passage through the nozzle [4]. The flow behavior index (n) values were below l for all samples (Table 1), further confirming their pseudoplastic behavior [17].

The structural recovery behavior of the samples during the 3ITT is shown in Fig. 3D. G′ decreased during the high-strain interval and subsequently increased when the strain was returned to 0.01%, indicating partial recovery of the gel structure after large deformation [18]. The recovery ratio decreased significantly with increasing oleogel proportion, and H_7_O_3_ exhibited the highest value among the biphasic bigel formulations (Table 3). These results suggest that a higher hydrogel proportion favored structural rebuilding after deformation, whereas increasing the oleogel fraction reduced the extent of structural recovery. Collectively, the rheological results indicate that increasing the oleogel proportion increased flowability but reduced elastic strength and structural recovery, which could affect shape retention after deposition.

**3D Printing performance and dimensional fidelity.** Printing performance was evaluated by comparing the appearance and dimensions of the printed constructs with those of the 15 × 15 × 10 mm CAD model (Fig. 4 and Table 4). All samples could be extruded and deposited, but their surface appearance and dimensional fidelity varied with the hydrogel-to-oleogel ratio. The printed surfaces appeared visually smoother as the oleogel proportion increased, whereas the width and length generally increased. The width and length of H_5_O_5_, H_3_O_7_, and H_0_O_10_ exceeded the corresponding design values, indicating increased lateral spreading. This behavior was consistent with the lower apparent viscosity, viscoelastic moduli, and recovery ratios of the oleogel-rich formulations, suggesting a reduced ability to resist deformation and rebuild the structure after deposition [4, 5]. Notably, H_7_O_3_ and H_5_O_5_ exhibited mean yield stress values of 142.19 and 101.82 Pa, respectively, although the difference was not statistically significant (Table 2). In contrast, H_7_O_3_ showed a significantly higher structural recovery ratio than H_5_O_5_ (88.7 vs 76.6%; Table 3). During layer-by-layer deposition, resistance to deformation and rapid structural recovery after extrusion are important for maintaining the deposited geometry. Consistent with these rheological characteristics, H_7_O_3_ exhibited limited lateral spreading and maintained dimensions close to the CAD model, whereas H_5_O_5_ showed greater lateral spreading after deposition. These results suggest that the superior dimensional fidelity of H_7_O_3_ was associated more clearly with its greater structural recovery than with yield stress alone.

In contrast, H_10_O_0_ produced a rough and uneven surface, with width and length values smaller than the corresponding CAD dimensions. Its relatively high apparent viscosity and resistance to deformation may have hindered uniform extrusion, contributing to the rough surface and dimensions smaller than those of the CAD model [4]. The printed height did not follow the same monotonic trend as the width and length, suggesting that vertical dimensional fidelity was not governed solely by lateral spreading. Overall, these results indicate that sufficient resistance to deformation and structural recovery were important for limiting lateral spreading, whereas excessive resistance to flow could hinder uniform extrusion. Thus, H_7_O_3_ provided the most favorable balance between visual surface uniformity and lateral dimensional fidelity among the biphasic formulations tested.

### Effects of PPI Concentration on the Thermal and Post-Heating Properties

Based on its high LBC, favorable structural recovery, and relatively high lateral dimensional fidelity, H_7_O_3_ was selected as the base formulation for evaluating the effects of PPI concentration on initial printing performance and post-heating stability.

**Temperature-dependent rheological properties.** The temperature-dependent changes in the complex shear modulus (G*) of H_7_O_3_ bigels containing different PPI concentrations during heating and subsequent cooling are shown in Fig. 5. During heating, G* gradually decreased in all formulations up to approximately 55°C, followed by a more pronounced decrease between approximately 55 and 63°C, indicating thermal softening of the bigel matrix.

Above approximately 63°C, the G* of the 0% PPI formulation continued to decrease, reaching approximately 10^2^ Pa at 80°C. In contrast, the PPI-containing formulations maintained values on the order of 10^3^ Pa, and the 7 and 10% PPI formulations exhibited a secondary increase in G*. This secondary increase may be associated with heat-induced denaturation and aggregation of potato proteins, particularly patatin. Patatin-rich potato proteins have been reported to undergo thermal denaturation at approximately 58–60°C, with the development of viscoelastic gel structures occurring at temperatures around 64°C [19]. Thermal unfolding can expose hydrophobic regions and promote intermolecular association, and hydrophobic interactions and hydrogen bonding have been identified as major contributors to potato protein aggregation [20, 21]. At higher PPI concentrations, the resulting heat-induced protein network may therefore have provided additional structural reinforcement as the bigel matrix softened during heating, partially compensating for the loss of matrix rigidity and contributing to the secondary increase in G*. Overall, PPI incorporation, particularly at 7 and 10%, improved the retention of rheological strength at elevated temperatures. Consistent with these changes in G*, tan δ increased around 55–65°C but remained below 1 throughout the heating range, indicating thermal softening without a transition to viscous-dominant behavior.

During subsequent cooling, G* increased in all formulations and rose sharply below approximately 40-45°C, reaching values on the order of 10^6^ Pa at 25°C. These results demonstrate pronounced cooling-induced stiffening of the bigel matrix. Because this increase was also observed in the 0% PPI formulation, the cooling response appeared to reflect restructuring of the overall bigel system rather than an effect attributable solely to PPI. During cooling, tan δ decreased as the temperature was lowered, particularly below approximately 45–50°C, while G* increased markedly, indicating progressive recovery and strengthening of the gel structure. No tan δ crossover above 1 was observed during either heating or cooling.

**Initial printing performance and post-heating dimensional stability.** The printing performance of H_7_O_3_ bigels containing different PPI concentrations was evaluated before and after heating (Figs. 6 and 7). Before heating, all formulations could be successfully extruded and deposited, producing self-supporting multilayer constructs. However, their dimensional accuracy varied with PPI concentration.

The 0% PPI formulation exhibited the lowest dimensional accuracy in length, width, and height. PPI incorporation significantly improved dimensional fidelity in all three directions. The 5 and 7% PPI formulations showed the highest length accuracy, whereas the 5-10% PPI formulations exhibited similarly high width accuracy. Height accuracy was also significantly higher in all PPI-containing formulations than in the 0% PPI formulation. These results indicate that PPI incorporation enhanced the ability of the printed constructs to retain their intended dimensions after deposition. This improvement was consistent with the higher initial G* values of the PPI-containing formulations, which may have increased resistance to deformation. However, the response was not strictly concentration-dependent, suggesting that initial printing accuracy was governed by a balance between extrusion flow and structural stability after deposition [4].

A heating condition of 120°C was selected as a standardized dry-heat treatment relevant to thermally processed meat products and 3D-printed meat systems [22]. Oven cooking approximately 120°C has been applied to meat products [22], and a heating condition of 120°C for 10 min has also been used for post-print cooking of 3D-printed beef products [23]. After heating at 120°C for 10 min, the 0% PPI construct lost its structural integrity and collapsed, whereas all PPI-containing constructs retained identifiable 3D shapes. The 3–7% PPI formulations exhibited lateral expansion accompanied by a reduction in height. However, the extent of lateral deformation progressively decreased with increasing PPI concentration. Length and width retention changed from approximately 123-127% in the 3% PPI formulation to approximately 95% in the 10% PPI formulation, with the latter showing values closest to the original dimensions. Height retention also increased at higher PPI concentrations, from approximately 60-64% in the 3 and 5% formulations to approximately 75−78% in the 7 and 10% formulations.

The improved post-heating dimensional stability at higher PPI concentrations was consistent with the greater G* retained by these formulations during heating. Overall, PPI incorporation improved both initial dimensional fidelity and post-heating shape retention. The 5 and 7% PPI formulations showed the highest initial lateral accuracy, whereas the 10% PPI formulation exhibited the greatest resistance to lateral deformation after heating.

**Cooking loss.** The cooking loss of the printed constructs after heating is shown in Fig. 8. Cooking loss could not be reliably determined for the 0% PPI formulation because the construct lost its structural integrity, collapsed, and spread during heating, preventing consistent recovery and weighing of the remaining sample. Among the PPI-containing formulations, cooking loss decreased from 22.75% in the 3% PPI formulation to 12.89% in the 10% PPI formulation. The 7 and 10% PPI formulations exhibited significantly lower cooking loss than the 3 and 5% PPI formulations (*p* < 0.05). The lower cooking loss at higher PPI concentrations may be associated with heat-induced structural reinforcement involving potato protein, which could reduce the release of liquid components from the bigel matrix during heating [24].

## Conclusion

This study investigated whether the room-temperature printability and post-heating stability of food-grade bigels could be sequentially controlled by adjusting the hydrogel-to-oleogel ratio and incorporating PPI as a heat-responsive component. The hydrogel-to-oleogel ratio significantly influenced liquid binding, rheological behavior, structural recovery, and dimensional fidelity. Among the biphasic formulations, H_7_O_3_ exhibited the highest liquid-binding capacity and structural recovery ratio and produced width and length values closest to those of the CAD model. H_7_O_3_ was therefore selected as the printable base formulation for PPI incorporation.

PPI incorporation improved the initial dimensional fidelity of the H_7_O_3_ constructs and reduced the extent of rheological softening during heating. The higher-PPI formulations, particularly those containing 7 and 10% PPI, retained higher G* values at elevated temperatures. During subsequent cooling, all formulations exhibited pronounced stiffening, indicating a strong temperature-dependent restructuring response of the bigel matrix. The rheological behavior observed during heating was reflected in the post-heating performance of the printed constructs. Whereas the 0% PPI constructs lost its structural integrity during heating, the PPI-containing constructs retained identifiable 3D shapes, and lateral deformation progressively decreased as the PPI concentration increased. The 10% PPI formulation showed width and length retention values closest to 100%, while the 7 and 10% PPI formulations exhibited significantly lower cooking loss than the 3 and 5% formulations.

Collectively, these results demonstrate that the hydrogel-to-oleogel ratio established the balance between extrusion and initial shape retention, whereas PPI concentration modulated the initial printing performance and post-heating stability of the printed bigels. Further studies should investigate the incorporation of these bigels into protein-based food matrices and evaluate their interfacial compatibility, storage stability, and sensory performance. Overall, the findings provide a basis for developing 3D-printable structured lipid systems with improved post-heating stability for potential fat-replacement applications in thermally processed plant-based foods.

## Figures and Tables

**Fig. 1:**
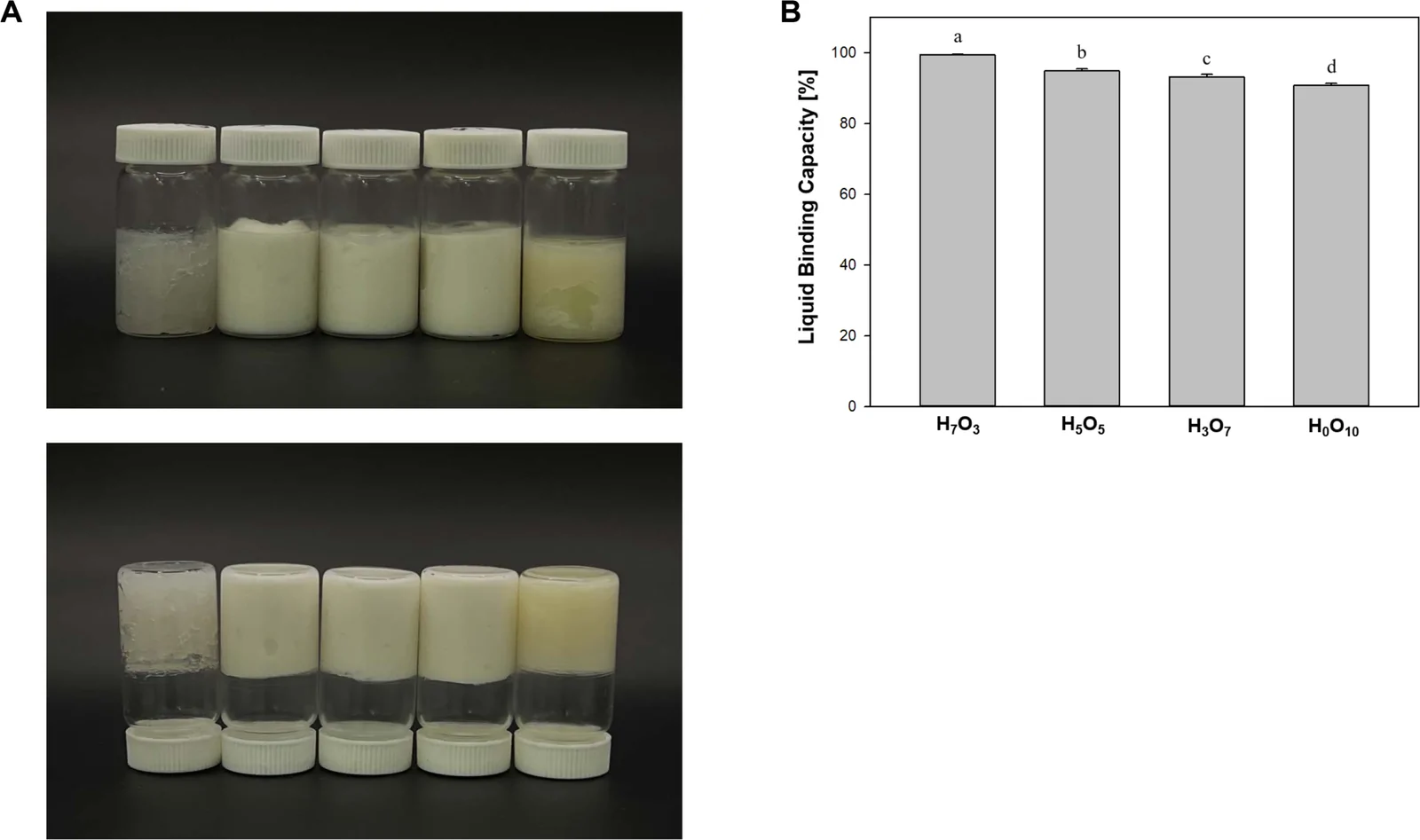
Physical stability and liquid binding capacity (LBC) of samples with different hydrogel-to-oleogel ratios.

**Fig. 2:**
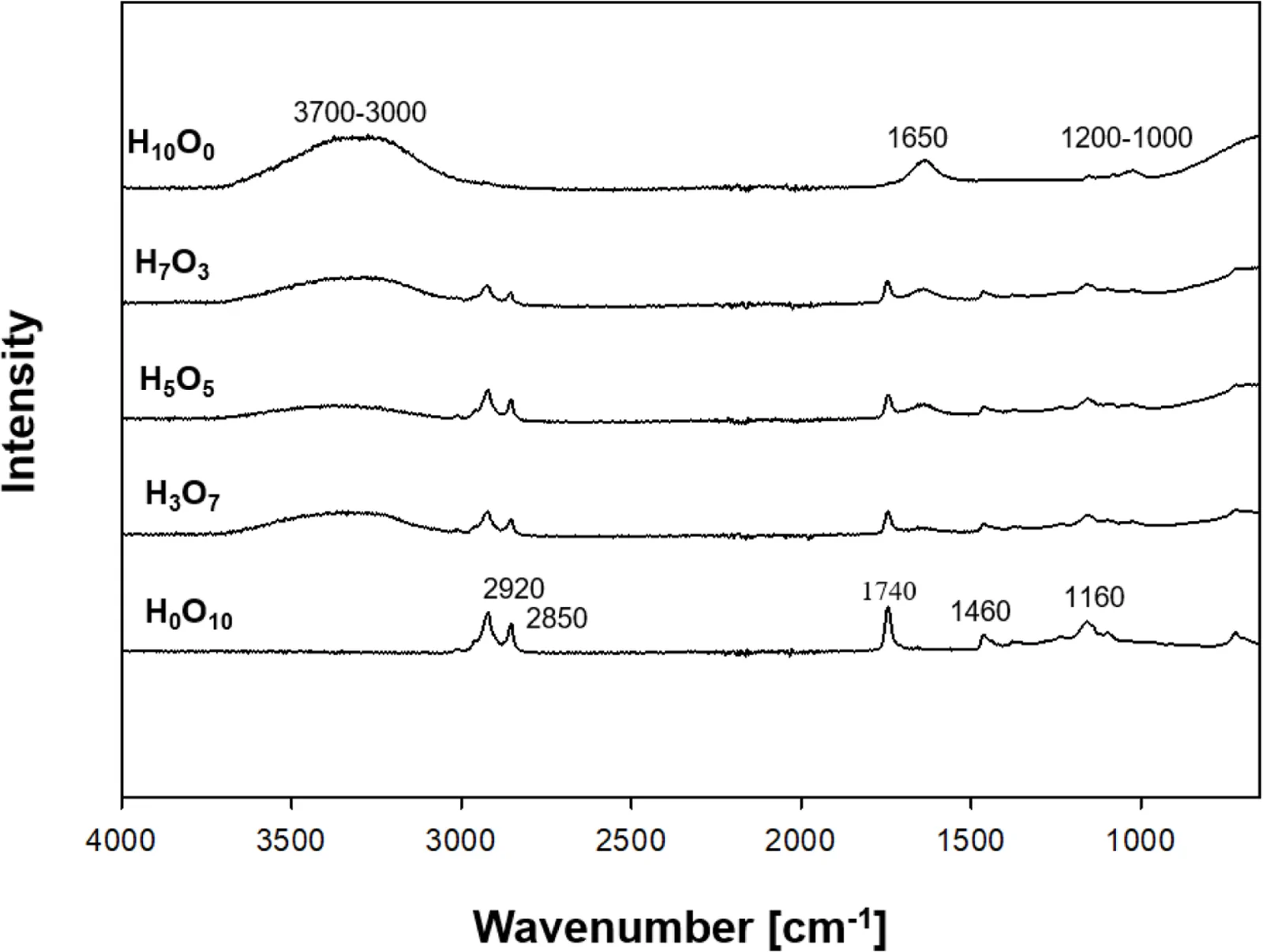
FT-IR spectra of samples with different hydrogel-to-oleogel ratios.

**Fig. 3:**
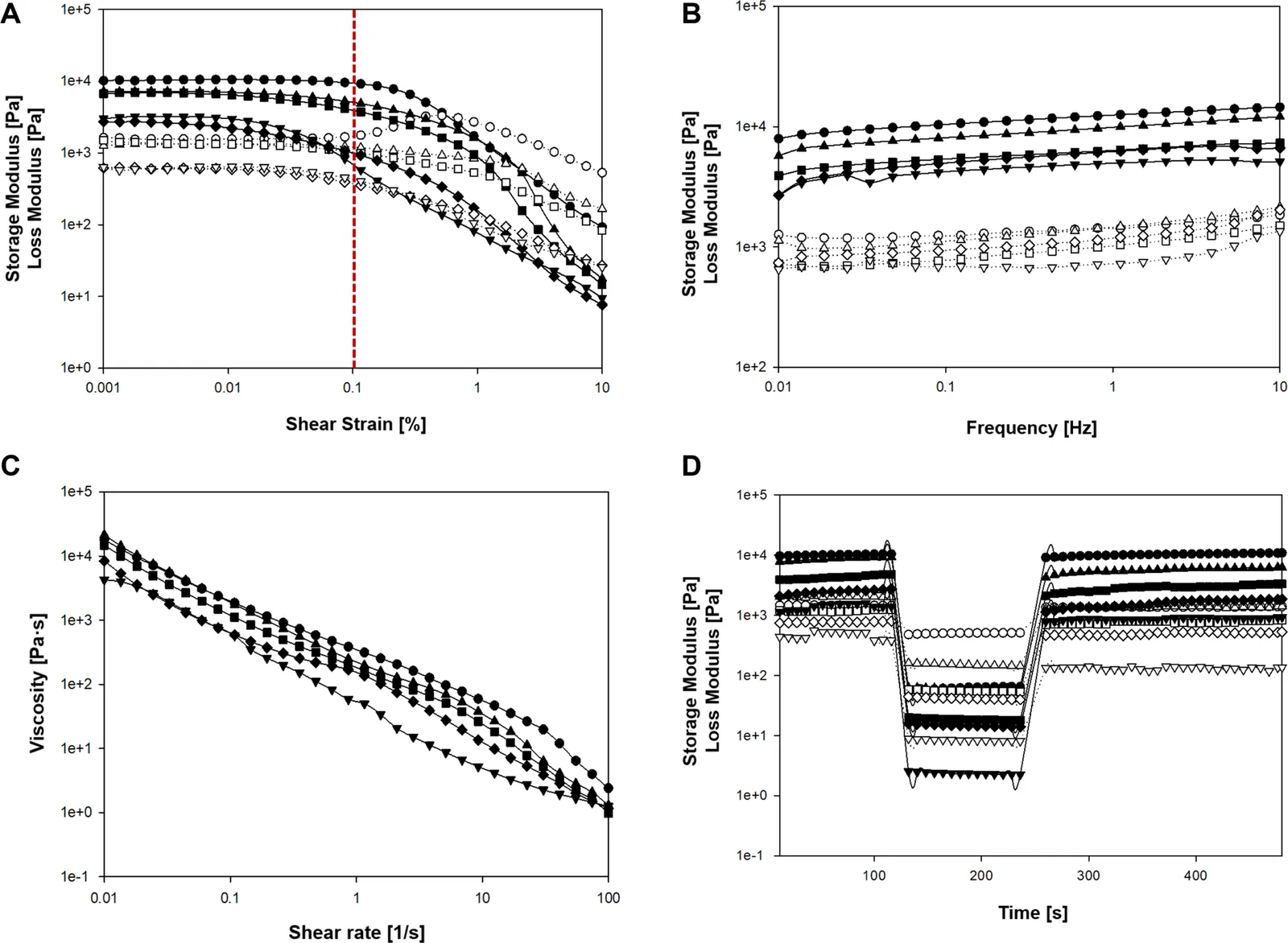
Rheological properties of samples with different hydrogel-to-oleogel ratios: (**A**) strain sweep, (**B**) frequency sweep, (**C**) flow curve, and (**D**) three-interval thixotropy test. H_10_O_0_ (●, ○), H_7_O_3_ ( ▲, △), H_5_O_5_ ( ■, □ ), H_3_O_7_ (◆, ◇), and H_0_O_10_ ( ▼, ▽). Filled and open symbols represent G′ and G″, respectively.

**Fig. 4:**
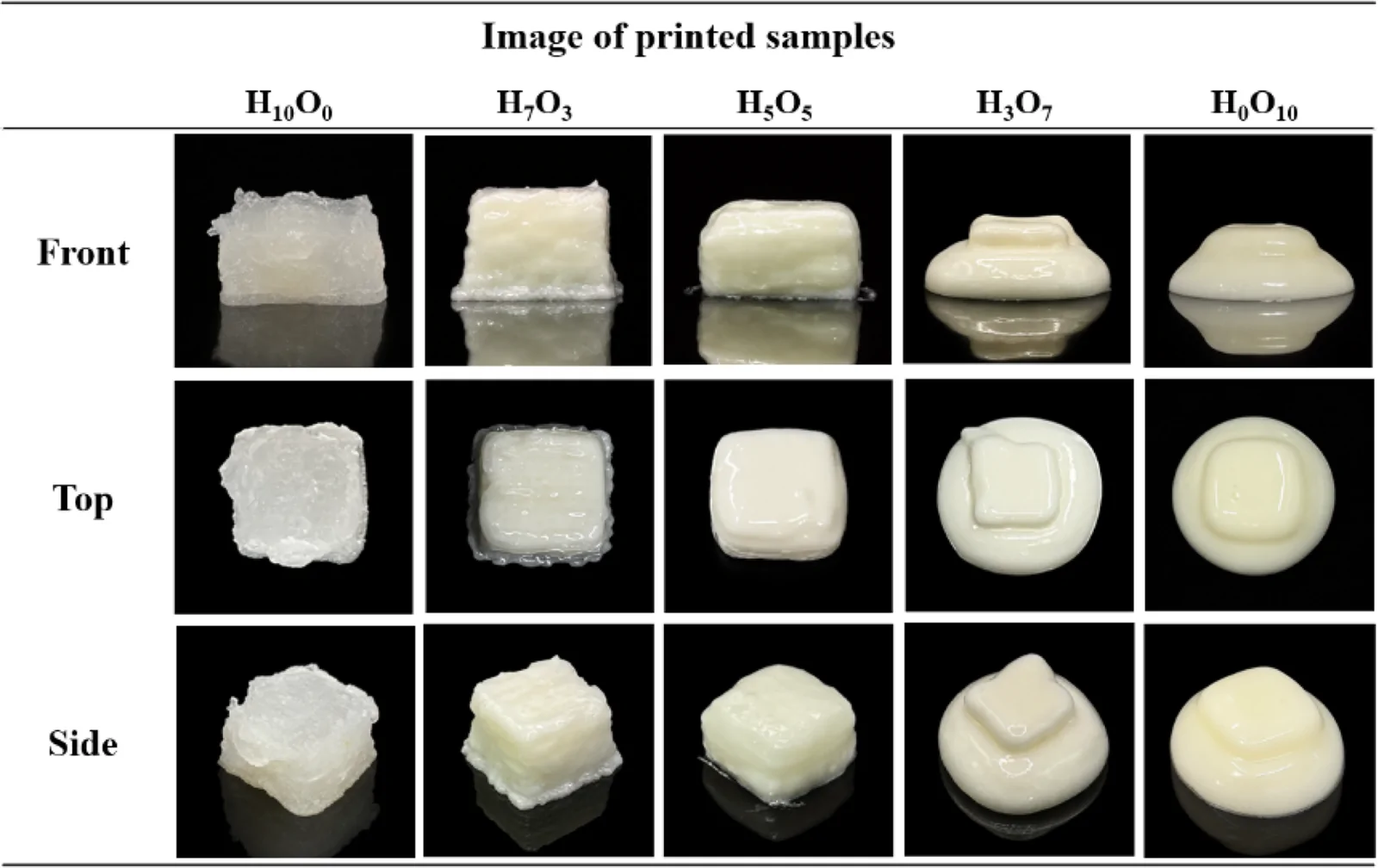
Representative front, top, and side views of 3D-printed constructs prepared from samples with different hydrogel-to-oleogel ratios.

**Fig. 5:**
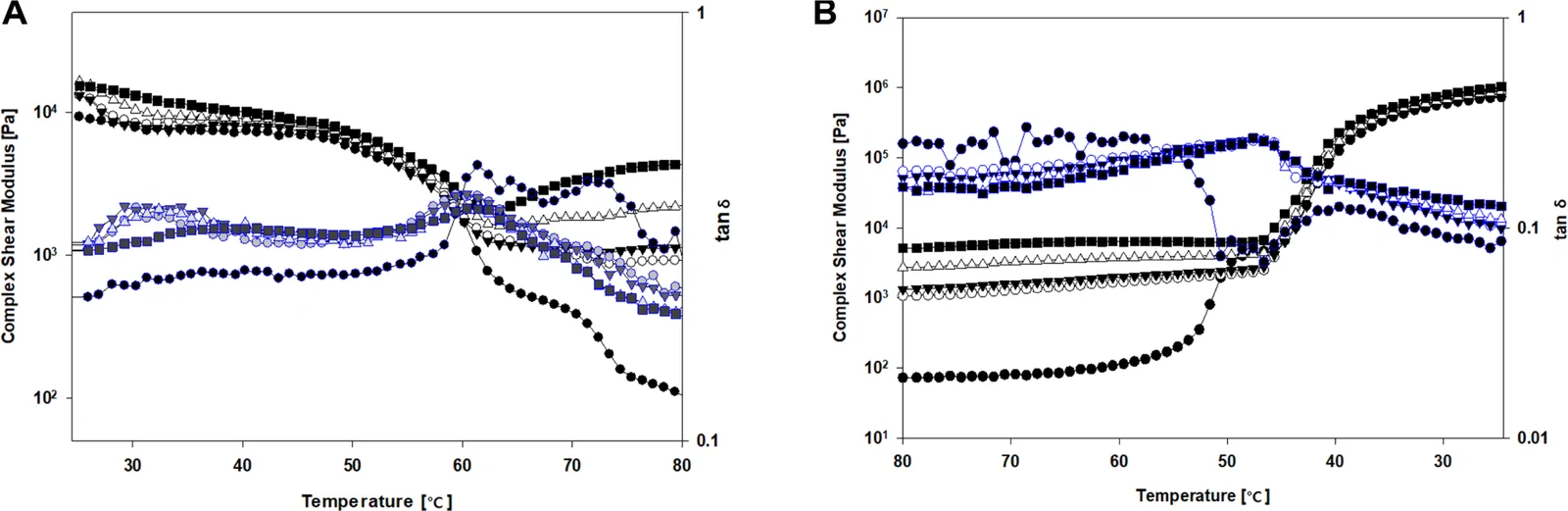
Temperature-dependent complex shear modulus (G*) and loss tangent (tan δ) of H_7_O_3_ bigels containing different concentrations of potato protein isolate (PPI) during heating from 25 to 80°C and subsequent cooling from 80 to 25°C. ●, ○, ▼, △, and ■ represent PPI concentrations of 0, 3, 5, 7, and 10%, respectively. Black and blue symbols represent G* and tan δ, respectively.

**Fig. 6:**
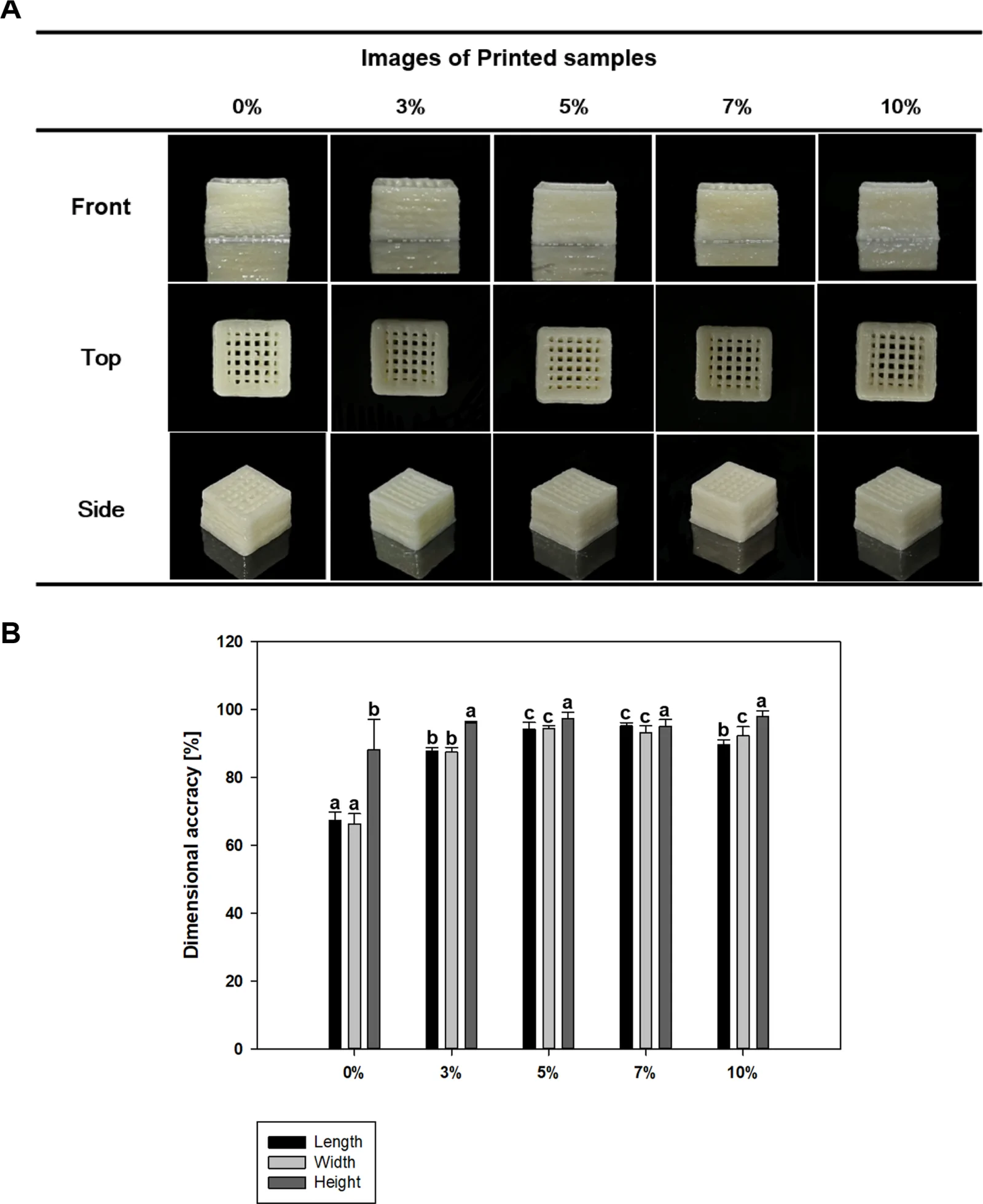
Initial printing performance of H_7_O_3_ bigels containing different concentrations of potato protein isolate (PPI).

**Fig. 7:**
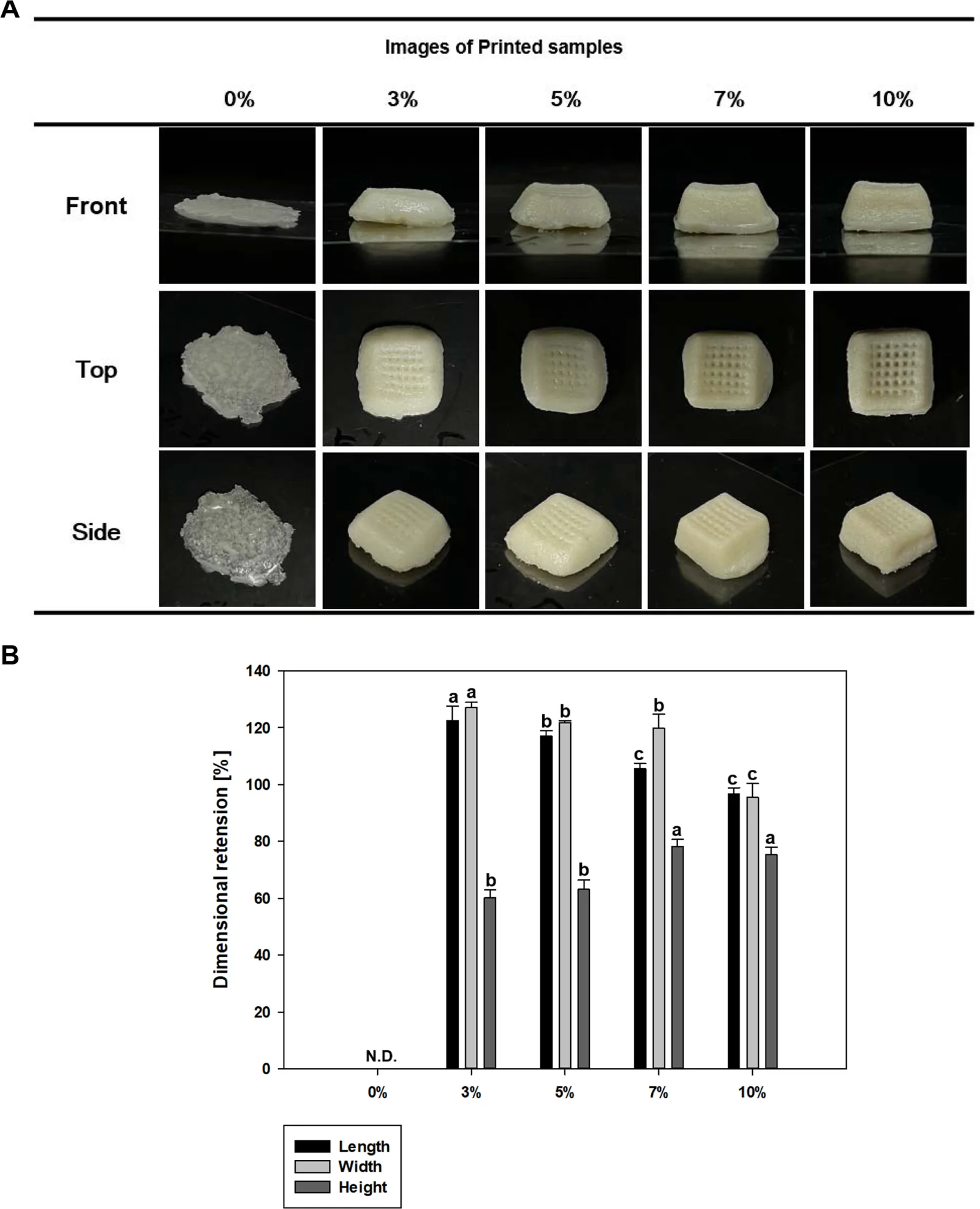
Post-heating dimensional stability of H_7_O_3_ bigels containing different concentrations of potato protein isolate (PPI).

**Fig. 8:**
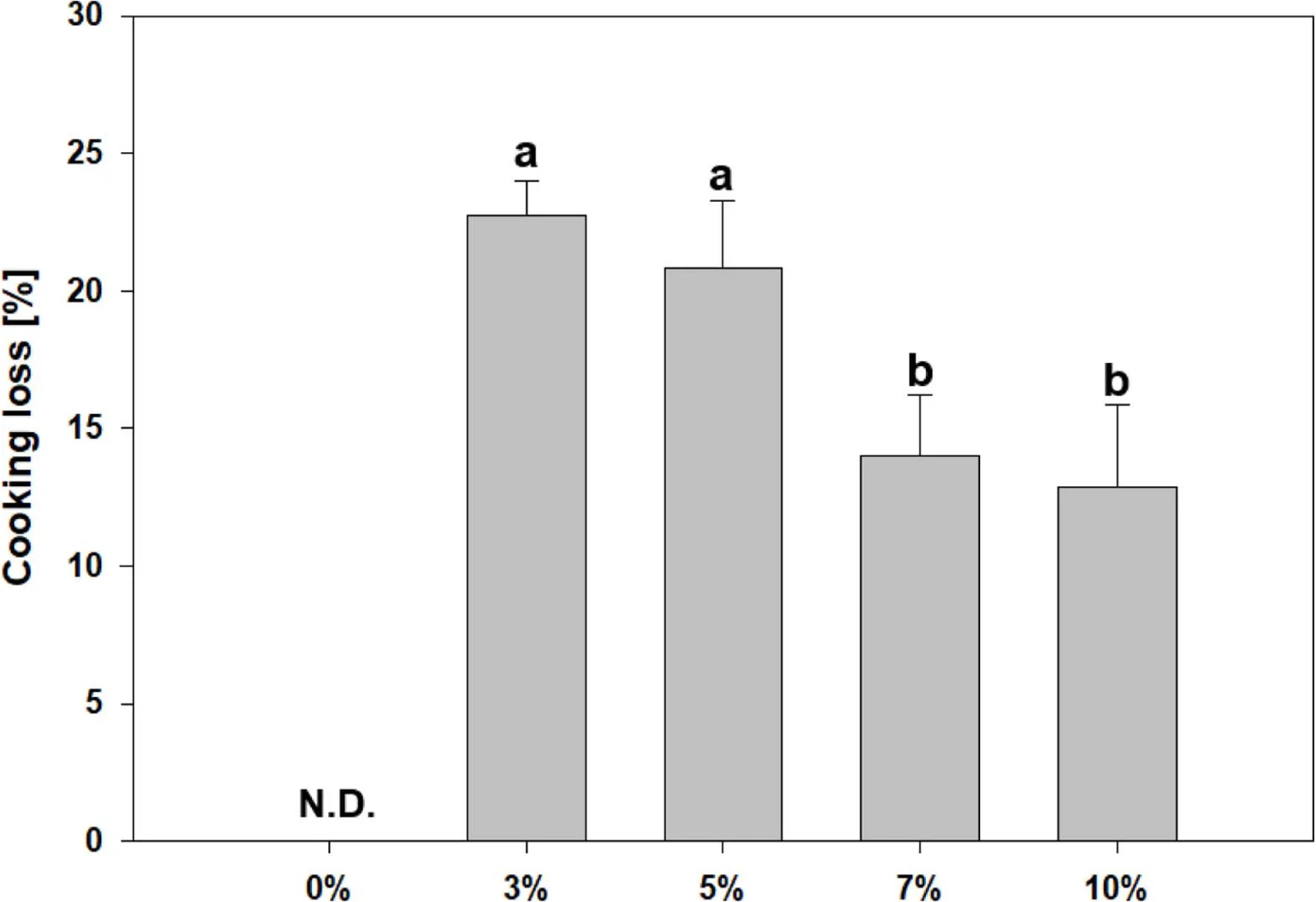
Cooking loss of 3D-printed H_7_O_3_ bigels containing different concentrations of potato protein isolate (PPI) after heating at 120°C for 10 min.

**Table 1:** Slope values of log G′ versus log frequency for samples with different hydrogel-to-oleogel ratios.

Sample	Frequency Sweep Test Slopes
H_10_O_0_	0.086 ± 0.019^a^
H_7_O_3_	0.096 ± 0.018^a^
H_5_O_5_	0.087 ± 0.027^a^
H_3_O_7_	0.116 ± 0.075^a^
H_0_O_10_	0.070 ± 0.029^a^

Values are expressed as mean ± SD (*n* = 3). The slope was obtained by linear regression of log G′ against log frequency. Different superscript lowercase letters within the same column indicate significant differences among the samples at *p* < 0.05 (one-way ANOVA followed by Duncan’s multiple range test).

**Table 2:** Consistency coefficient (K), flow behavior index (n), yield stress, and coefficient of determination (R^2^) of samples with different hydrogel-to-oleogel ratios.

Sample	K (× 10^5^ Pa·s^*n*^)	*n*	Yield stress (Pa)	*R* ^2^
H_10_O_0_	2.47 ± 0.02^a^	0.14 ± 0.01^a^	167.66 ± 9.18^a^	0.94 ± 0.02^a^
H_7_O_3_	2.28 ± 0.07^b^	0.11 ± 0.08^a^	142.19 ± 40.30^ab^	0.96 ± 0.02^a^
H_5_O_5_	2.18 ± 0.02^b^	0.13 ± 0.08^a^	101.82 ± 31.83^b^	0.94 ± 0.03^a^
H_3_O_7_	2.11 ± 0.12^b^	0.10 ± 0.01^a^	99.29 ± 27.75^b^	0.96 ± 0.01^a^
H_0_O_10_	1.64 ± 0.15^c^	0.18 ± 0.13^a^	29.83 ± 24.12^c^	0.95 ± 0.05^a^

Values are expressed as mean ± SD (*n* = 3). Different superscript lowercase letters within the same column indicate significant differences among the samples at *p* < 0.05 (one-way ANOVA followed by Duncan’s multiple range test).

**Table 3:** Recovery ratios of samples with different hydrogel-to-oleogel ratios determined by a three-interval thixotropy test (3ITT).

Sample	H_10_O_0_	H_7_O_3_	H_5_O_5_	H_3_O_7_	H_0_O_10_
Recovery	98.4 ± 4.8^a^	88.7 ± 3.2^b^	76.6 ± 7.1^c^	63.3 ± 5.1^d^	71.1 ± 3.6^cd^

Values are expressed as mean ± SD (*n* = 3). The recovery ratio was calculated as the ratio of G measured at the end of the third interval to that measured at the end of the first interval. Different superscript lowercase letters within the same row indicate significant differences among the samples at *p* < 0.05 (one-way ANOVA followed by Duncan’s multiple range test).

**Table 4:** Dimensions of 3D-printed constructs prepared from samples with different hydrogel-to-oleogel ratios.

Sample	Width (mm)	Length (mm)	Height (mm)
CAD	15.00 ± 0.00^d^	15.00 ± 0.00^c^	10.00 ± 0.00^c^
H_10_O_0_	12.70 ± 0.27^f^	13.48 ± 0.47^d^	9.81 ± 0.17^c^
H_7_O_3_	14.13 ± 0.26^e^	14.63 ± 0.42^c^	12.27 ± 0.18^a^
H_5_O_5_	18.52 ± 0.42^c^	18.83 ± 0.17^b^	11.43 ± 0.22^b^
H_3_O_7_	23.73 ± 0.59^b^	27.75 ± 0.36^a^	11.40 ± 0.34^b^
H_0_O_10_	28.17 ± 0.57^a^	28.12 ± 0.60^a^	11.78 ± 0.49^ab^

Measured values are expressed as mean ± SD (*n* = 3). CAD values indicate the designed dimensions and were not included in the statistical analysis. Different superscript lowercase letters within the same column indicate significant differences among the printed samples at *p* < 0.05 (one-way ANOVA followed by Duncan’s multiple range test).
